# Visualization of air flow around soccer ball using a particle image velocimetry

**DOI:** 10.1038/srep15108

**Published:** 2015-10-08

**Authors:** Sungchan Hong, Takeshi Asai, Kazuya Seo

**Affiliations:** 1 Institute of Health and Sports Science, University of Tsukuba, Tsukuba, 305-8574, Japan; 2 Department of Education, Art and Science, Yamagata University, Yamagata, 990-8560, Japan

## Abstract

A traditional soccer ball is constructed using 32 pentagonal and hexagonal panels. In recent years, however, the likes of the Teamgeist and Jabulani balls, constructed from 14 and 8 panels, respectively, have entered the field, marking a significant departure from conventionality in terms of shape and design. Moreover, the recently introduced Brazuca ball features a new 6-panel design and has already been adopted by many soccer leagues. However, the shapes of the constituent panels of these balls differ substantially from those of conventional balls. Therefore, this study set out to investigate the flight and aerodynamic characteristics of different orientations of the soccer ball, which is constructed from panels of different shapes. A wind tunnel test showed substantial differences in the aerodynamic forces acting on the ball, depending on its orientation. Substantial differences were also observed in the aerodynamic forces acting on the ball in different directions, corresponding to its orientation and rotation. Moreover, two-dimensional particle image velocimetry (2D-PIV) measurements showed that the boundary separation varies depending on the orientation of the ball. Based on these results, we can conclude that the shape of the panels of a soccer ball substantially affects its flight trajectory.

In general, the panel shapes and design of the official FIFA ball are changed when the World Cup is held, which is once every four years. The balls used in the early years of the World Cup were constructed from panels that were identical to those of volleyballs. As there was no official ball at that time, matches were played with the national teams’ own balls. Notably, the final of the first World Cup between Argentina and Uruguay was played with each team playing one half of the game with their own ball. The first ever official ball (Telstar, 32 panels, Adidas) was used for the Mexico World Cup in 1970. Thereafter, and until the South Korea/Japan World Cup of 2002, the official ball (Fevernova, 32 panels, Adidas) was constructed from panels of a design that had not changed for approximately 30 years. The characteristic feature of the official ball of that time was its construction from 32 pentagonal and hexagonal panels. Subsequently, after a new official ball (Teamgeist, 14 panels, Adidas) was used for the Germany World Cup in 2006, the configuration and shapes of the panels have evolved substantially. The Teamgeist ball, the official ball in 2006, did not use the 32 conventional (hexagonal and pentagonal) panels. Instead, it introduced a much-debated, innovative shape with 14 panels, and became the subject of numerous aeromechanical studies^1^,^2^,^3^,^4^,^5^,^6^. Subsequently, the Brazuca ball (six panels, Adidas), a further evolution, made its appearance at the 2014 Brazil World Cup. With the soccer ball evolving in this manner, a variety of aeromechanical studies have been conducted on 14-, 8- and 6-panel balls, as well as on the conventional 32-panel ball^7^,^8^,^9^,^10^,^11^,^12^. In a more recent study, trajectory analyses and wind tunnel experiments were performed to glean information for comparing the non-spin aerodynamics of different soccer balls^13^.

However, there have been no studies of the flow of air around a soccer ball with respect to the shape, number, and orientation of the ball’s panels. The subject of our experiment was a soccer ball known as the Cafusa. We examined the air flow over the surface of the ball using particle image velocimetry (PIV). A characteristic of the Cafusa ball is the manner in which the shapes of its panels change significantly depending on the orientation of the ball (more so than other balls). Therefore, the goal of our experiment with the Cafusa ball was to examine the effects of the panels (number of panels, interval, etc.). Moreover, this study focused on visualizing the flow over three orientations (faces) of the Cafusa ball through the application of two-dimensional particle image velocimetry (2D-PIV). Furthermore, we attempted to clarify the effect of the panel shapes, which differ depending on the orientation of the ball, on the ball’s trajectory and the flow of the air around the ball.

Based on the observed 2D-PIV results and actual ball trajectories, we clarified how the panel characteristics affect the flight of a soccer ball, which enables the prediction of its trajectory.

## Results

### Variation in drag coefficient with orientation of ball

**Figure 1:**
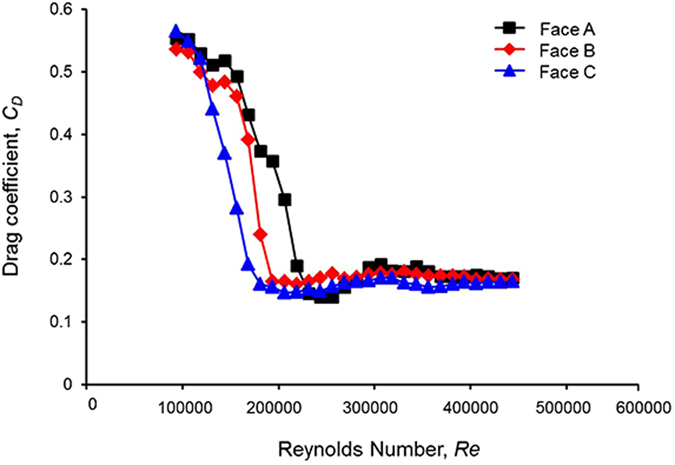
Variation in drag coefficient corresponding to ball orientation (Faces A–C).

Figure 1 shows the Cafusa ball’s drag coefficient for each orientation (Face A, B, C). The graph shows that the critical drag range, which represents a significant change in the drag, is the lowest for face C, followed by faces B and A, in that order. Moreover, we observed that *Cd* decreased from approximately 0.5 to <0.2 for *Re* ≥ 1.5 × 10^5^ for faces A and B, and for *Re* ≥ 1.3 × 10^5^ for face C. The critical Reynolds numbers for A, B, and C correspond to *Cd* values of approximately 0.14, 0.16, and 0.15, respectively, with the value for face B being higher than those for the other faces. Moreover, we found that the drag on the ball varied as the orientation of the ball (its face) varied, even for the same ball.

### Side and lift forces in the wind tunnel

**Figure 2:**
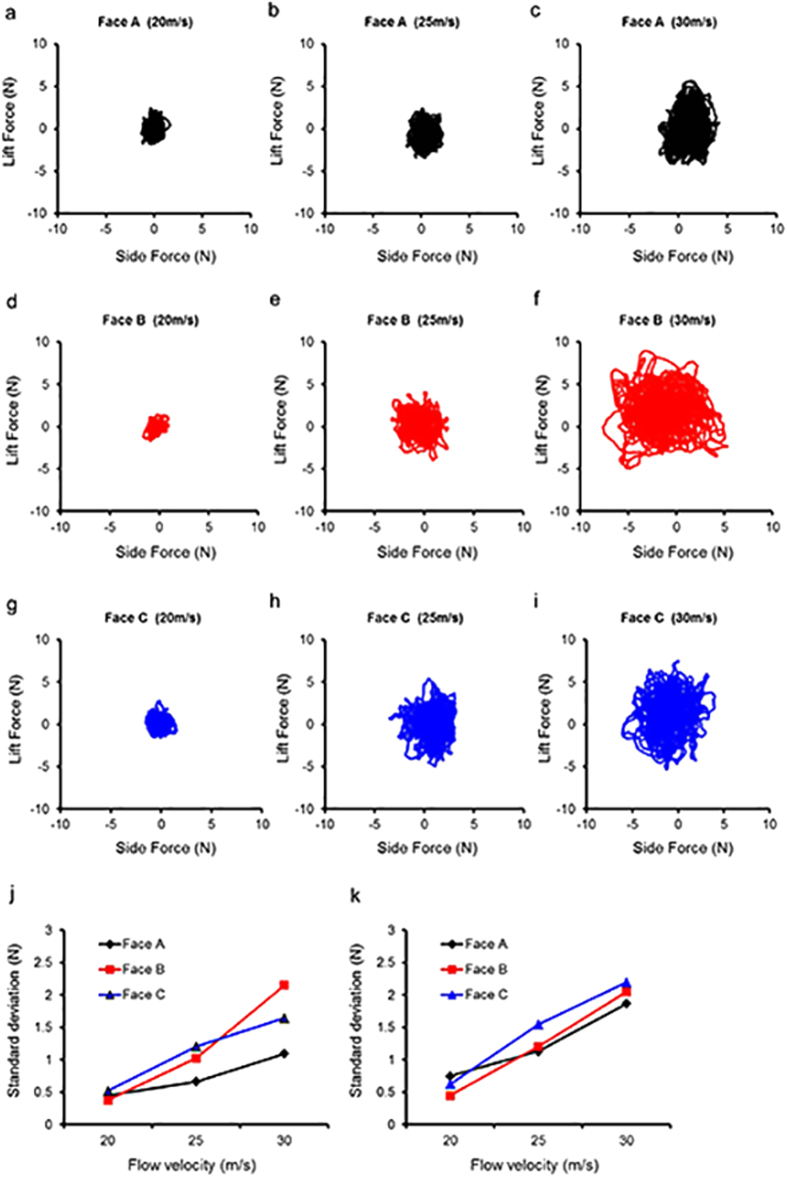
Scatter plots for side and lift forces of balls and *SD*s of the respective forces for each flow velocity (after 9 s). As the flow velocity increases from 20 m/s (**a,d,g**) to 30 m/s (**c,f,i**), the irregular fluctuations in the side and lift forces increase. The *SD* values of the side (**j**) and lift forces (**k**) increase with the flow velocity.

Figure 2 shows scatter plots of the lift and side forces experienced by the soccer ball for different panel orientations. These indicate that the irregular fluctuations increase as the flow velocity increases from 20 m/s to 30 m/s. The change in the irregular fluctuations as the speed increases is smaller for face A than for the other faces, while face B showed the greatest change. The *SD* of the side and lift forces also increases with the flow velocity (Fig. 2j,k).

In line with the findings of a previous study^13^, regarding the relationship between the lift and the side forces, the changes in the force increased with the acceleration. In the case of the Cafusa ball in particular, the change in the force in the lateral directions was more significant than that in the vertical direction, indicating that the lateral changes due to the panel orientation were greater than the vertical changes.

### Point of ball impact using kicking robot

**Figure 3:**
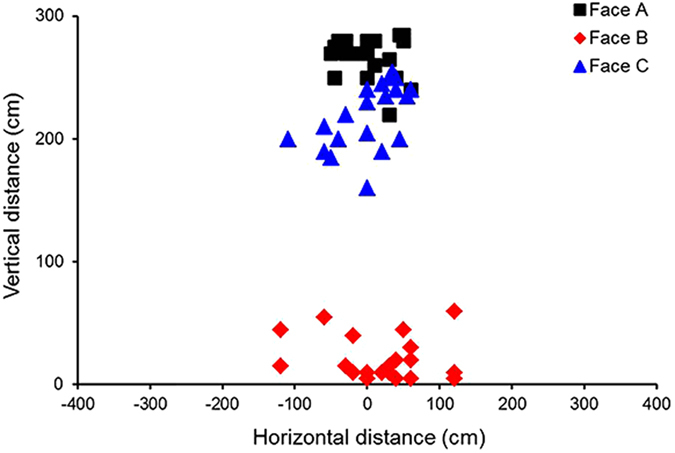
Impact points for each face of the Cafusa.

First, actual balls were launched by an impact-type kicking robot at a net 25 m away. The points at which the balls hit the net are plotted in the following Fig. 3 as points of impact. The balls were launched under the following conditions: an initial ball velocity of 30 m/s with less than one rotation (no rotation). This was repeated 20 times per panel orientation for each type of soccer ball. The Cafusa ball exhibited instability as the ball trajectory varied considerably according to the panel orientation. When we compared the standard deviations of the impact point, we found that the Cafusa ball exhibited standard deviations of 0.17 m, 0.39 m, and 0.19 m for the vertical direction, and 0.45 m, 0.68 m, and 0.38 m for the horizontal direction (faces A, B, and C, respectively). This indicates a relatively irregular flight trajectory for face B.

**Figure 4:**
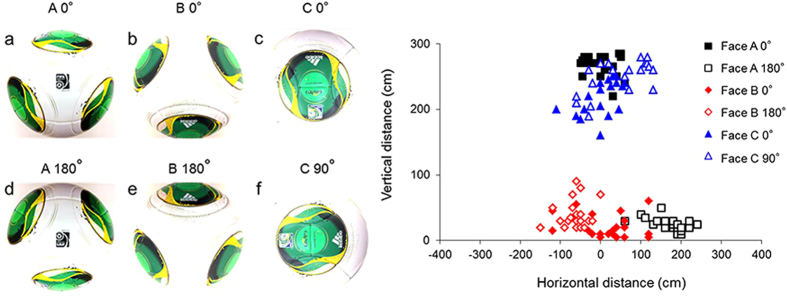
Comparison of variation in points of impact by rotation of panel orientation.

Next, Fig. 4 shows the graph comparing the points of impact of the same Cafusa ball when each of its panel orientation was rotated by 180° (90° for panel orientation C) and launched by the kicking robot. The points of impact with face A (Fig. 4a) shifted drastically from upper region to lower region by varying the angle of orientation by 180° (Fig. 4d). The graph shows that the flight trajectory (point of impact) of the actual ball with face B (Fig. 4b) shifted from right to left in relation to the centre line of the goal when it is rotated by 180° (Fig. 4e). Based on these results, we were able to state that the orientation of the ball produces extreme changes in its trajectory and significantly affects its flight characteristics.

### Velocity vectors on suction side of the ball

**Figure 5:**
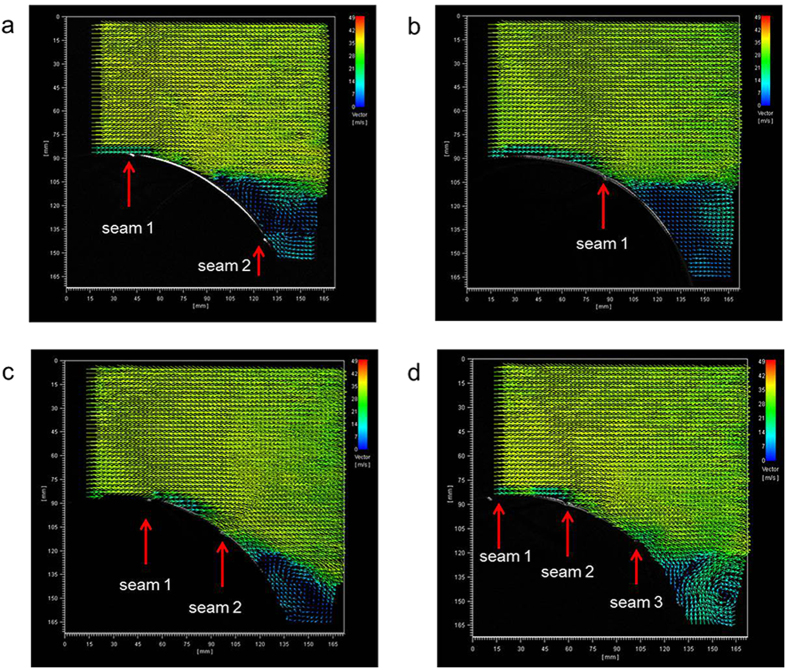
Velocity vectors on the suction side of the soccer balls at U = 30 m/s.

To understand the relationship between the orientation of the panels of the ball and the air flow around it, the flow of the air around the ball at a velocity of 30 m/s was examined using 2D-PIV (Fig. 5). For the Cafusa ball that was the subject of this experiment, the number of seams and their position on the surface (sagittal plane) vary greatly depending on the orientation of the ball. Therefore, we considered four different panel orientations. The positions of the separation points differed according to the position of the seams and their number in the sagittal plane of the soccer ball. Figure 5 shows the instantaneous vector fields for the positions of each seam. First, when there were two seams spaced about 80 mm apart (such that the angle formed with the stagnation point in seam 1 was 95°, and with that in seam 2 was 150°), the separation point was found to be at about 120°, (nearly 65 mm from the centre of the ball) (Fig. 5a). Further, for the orientation with a single seam (120° in seam 1), the separation point was at about 125° (about 70 mm from the centre of the ball), and was moved back a little (Fig. 5b). The reason for this is believed to be that if there is a seam at the position where a separation point occurs (at about 120°), then the flow of the air is moved back under the influence of the seam (Fig. 5a,b). Furthermore, in the face with two seams and a relatively narrow spacing of about 50 mm between them, (100° in seam 1, and 130° in seam 2), the seams affected the flow of the air and the separation point was moved to about 140° (about 85 mm) (Fig. 5c). The phenomenon observed here was caused by the flow of the air being separated once by the first seam (seam 1), but becoming re-attached immediately before being completely separated by the next seam (seam 2) (Fig. 5c). For the face with three seams, and the spacing between them being about 45 mm, (80° in seam1, 105° in seam 2, and 130° in seam 3), the air flow around the soccer ball re-attached at seams 2 and 3, and the separation point was at about 145° (about 90 mm), having moved to the rearmost position (Fig. 5d). This suggests that, even if there are seams in identical positions (130° seam), (seam 2 in Fig. 5c, seam 3 in Fig. 5d), the flow of the air varies depending on the relationship with the other seams (the number and spacing of the seams). From these results, we were able to determine that the separation point varies greatly depending on the orientation of the panels, even for identical soccer balls. Thus, it can be seen that the type of seam on the surface of the ball (number and spacing) changes the air flow around it and by actually acting upon its aerodynamics, affects the flight of the ball. Hence, the seams that make up the surface of the ball (number of seams, spacing, and position) can be said to be one of the factors that determine the trajectory of the ball. As this study only performed a two-dimensional analysis, in the future it will be necessary to perform a three-dimensional analysis of the structure of the seam to determine the relationship between the seam and the aerodynamics acting on the soccer ball in more detail.

### Lift forces and velocity vectors on suction side of a cylinder

**Figure 6:**
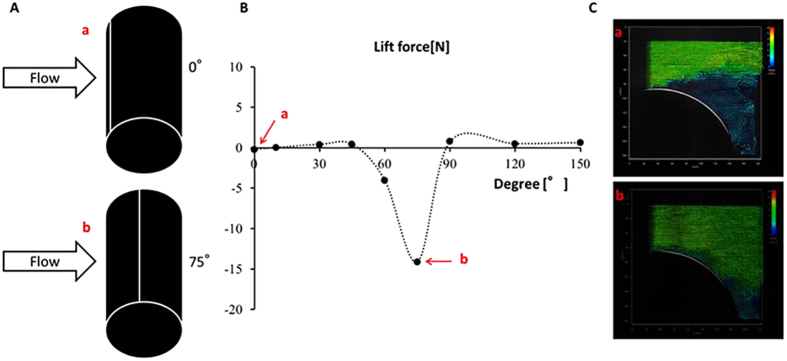
(A) Cylinder; (B) Lift force due to rotation of cylinder; (C) Comparison of velocity vectors at 0° and 75°.

Figure 6 shows the PIV results for a cylinder with a single groove (width 1 mm, depth 2 mm). The force of the air was measured using PIV while the aerodynamic forces were measured for an air flow velocity of 24 m/s for 5 s. The lift force was measured while rotating the single-grooved cylinder from 0° to 150° (Fig. 6A). Between groove orientations of 0° to 45°, a similar lift force (between −0.2 N to 0.4 N) was observed. However, when the position of the groove reached 60° a slightly downward lift force (−4.0 N) arose. The largest force (−14.1 N) was observed when the position of the groove was at 75°. When the groove was at 90° (at the very top of the cylinder) the associated lift force became small (0.7 N), and the force remained at a similar level until it reached 150° (0.5 N at 120° and 0.6 N at 150°) (Fig. 6B). Furthermore, from the PIV measurements, separation was observed to occur at about 100° when the groove was at 0°. As the groove was rotated, the separation point shifted backwards, up to about 150° for the case of the groove at 75°, where the lift force increased to a maximum (Fig. 6C). In this example, the effects of a single groove can be observed, and the force acting on the cylinder varies significantly depending on the position of the groove. It is believed that this result can be compared with that obtained with the Cafusa ball, and can explain the effects of the grooves between the panels on the surface of the soccer ball. In other words, the positions of the seams on the surface of the soccer ball are regarded as having a significant impact on the aerodynamic characteristics and trajectory. However, because the shapes of the panels that constitute the surface of the Cafusa ball are not identical and are configured in a complex manner, it is necessary to conduct a more detailed examination on the effects of the surface of the ball. Future work should include examining the relevance of the depth of the grooves, a cylinder with two grooves, and a cylinder with grooves at varying intervals.

Although, in this study, we examined the lateral air flow over the surface of a soccer ball, in subsequent work, we should examine the air flow over the entire surface of the ball. We also believe that it will be necessary to examine the relationship between the surface of the ball and separation, by examining, in detail, the vortex that occurs on the wake-flow side of the ball.

## Discussion

This study set out to examine the air flow over the surface of a ball by using PIV measurement, to observe the effects of the panels of a soccer ball, which were originally revealed in a previous study^11^,^13^. As a result, we found that the grooves between the panels on the surface of the ball have a significant impact on its aerodynamic characteristics. Furthermore, the positions, numbers, and even intervals of the grooves affect the separation point on the surface of a soccer ball, which has a significant impact on the aerodynamic characteristics and trajectory of the ball.

The subject of our experiment was the Cafusa soccer ball. We examined the air flow over the surface of the ball using PIV. The Cafusa is characterised by panels having shapes that change significantly depending on the orientation of the ball (more so than other balls). Therefore, our experiment with the Cafusa ball was performed to examine the effects of the panels (number of panels, interval, etc.).

A trend of the resistance changing with the orientation of the panels was observed from the results of our wind tunnel experiment. This change was particularly significant in the intermediate speed range for which the air flow velocity was 7 to 20 m/s, which led us to believe that the orientation of the panels would have a significant effect on actual passes and long kicks in a soccer match.

The results of the point of impact tests using a kicking robot also revealed that, depending on the orientation of the ball, the trajectory changed significantly. Furthermore, the variance resulting from the point of impact in the lateral direction (*SD* value) was greater with face B than with the other faces. According to the lift force results obtained from the wind tunnel experiment, the *SD* rate of increase for face B was 710%, this being the largest figure, and this was similar to the results derived from the point of impact experiment. Furthermore, the results obtained with the kicking robot are believed to explain why changes due to the panel orientation are greater in the lateral direction than in the vertical direction in the wind tunnel experiment. In other words, the actual trajectory could be predicted based on the results of the wind tunnel experiment on the panel orientation of the ball.

The 2D-PIV results led us to believe that changes in the positions of seams arising from changes in the orientations of the panels have a significant impact on the air flow around the ball. We were able to confirm that the separation point changes with the number of seams. Moreover, even for a panel orientation that has the same number of seams, the separation point can change depending on the panel intervals and the lift force. Furthermore, the PIV results from the experiment with a cylinder clearly revealed the effects of the seams on the surface, and the positions of the seams are believed to have a significant impact on the aerodynamics of the ball. This result can be extended to the grooves between the panels on the surface of a soccer ball and is believed to explain the effects of the seams of the ball. In other words, the shape of the surface of the ball, which depends on the orientation, is believed to have a significant impact on the aerodynamic characteristics and trajectory of the ball. However, the present study has taken the panel shape (including the panel intervals and panel numbers) into account in setting the seam location and has studied the airflow over the surface of the ball under conditions where larger differences can be observed. Therefore, future study is required for various other seam locations on the same panels. Moreover, we believe a study is also required on the effect on the airflow around the ball by the differences in the seam interval (differences in the distance to the seam) and the depth and width of the seams.

The change in the air flow over the surface of the ball, caused by the shapes of the panels, has an impact on the aerodynamic characteristics and trajectory of the ball, which supports the findings of previous studies^11^,^13^. Therefore, it is believed that, given these results, the aerodynamic characteristics and trajectory of a ball could be predicted based on the shapes of the panels. Furthermore, because an awareness of the effects of the panels of a soccer ball would be useful for coaching on the field, a contribution towards improved player performance should be possible. Clarifying the structures and principles of the balls used in a variety of sports would make it possible to acquire more efficient techniques that are supported by scientific evidence, which would contribute not only to the training of athletes aiming for peak performance but also in substantiating instruction methods for physical education in schools.

## Methods

### Wind tunnel tests

**Figure 7:**
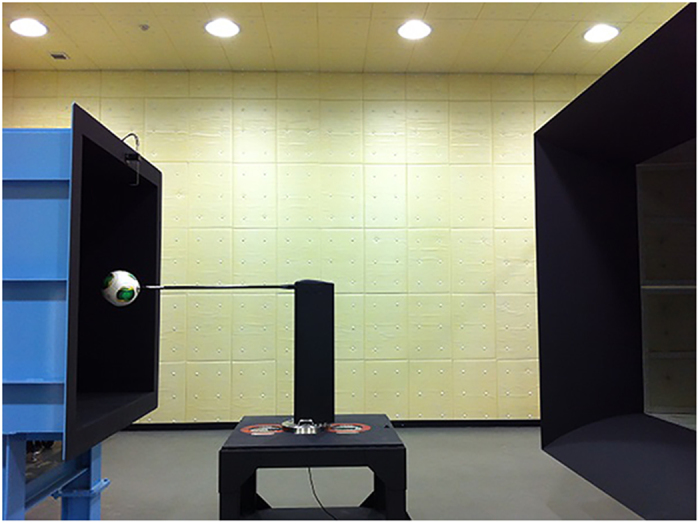
Setup of wind tunnel test.

**Figure 8:**
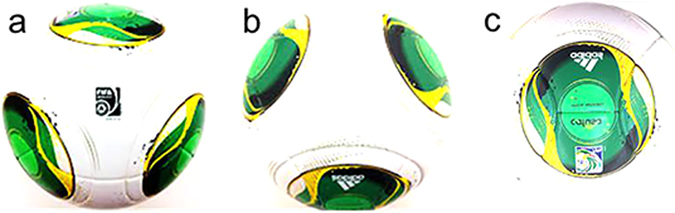
Ball orientations (face a–c).

For this experiment, we used the low-speed circular wind tunnel at Tsukuba University (San Technologies Co., Inc.), shown in Fig. 7. The maximum air flow velocity was 55 m/s, the test section size was 1.5 m × 1.5 m, the air flow velocity distribution was within ±0.1%, and the turbulence was 0.1% or less. An experiment was conducted in the wind tunnel using the new Cafusa soccer ball (32 panels, Adidas). For this experiment, we assumed three faces for the ball orientation (A, B, and C), and then measured the aerodynamic forces acting on each ball face (Fig. 8). Furthermore, to investigate the changes in the aerodynamics with the orientation of the ball as it rotates, we changed the position of the faces of identical panels from 0° to 360° at 90° intervals, and measured the aerodynamics with an air flow velocity (*U*) of between 7 m/s and 35 m/s. The faces were fixed as shown in Fig. 8, and the force acting on the soccer ball was measured using a sting-type six-component force sensor (LMC-61256, Nissho Electric Works). The aerodynamic forces measured in this experiment were converted into the drag coefficient (*Cd*), lift coefficient (*Cl*), and transverse force coefficient (*Cs*), as given by equations (1), (2), and (3).


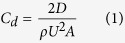



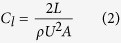



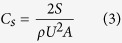


Where *ρ* is the air density (1.2 kg/m^3^), *U* is the flow rate, and *A* is the projected area of the ball given by *A* = *π* × 0.112 m = 0.038 m^2^.

### Point of impact test for soccer balls using a kicking robot

**Figure 9:**
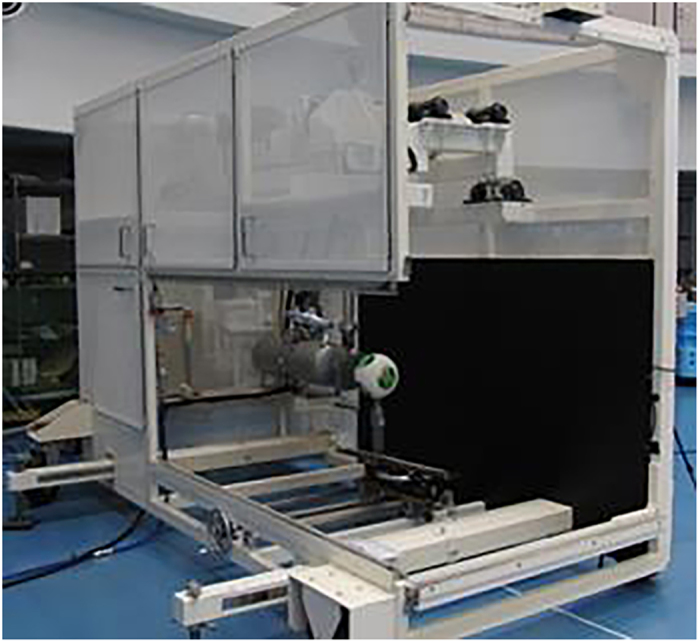
Multipurpose kicking robot.

The experiment was conducted indoors, in the Sport Performance and Clinic Laboratory of Tsukuba University, and therefore was not affected by wind. A full-size soccer goal was placed 25 m in front of the kicking robot, which kicked a non-rotating ball aimed at the centre of the goal (Fig. 9). A stationary ball was placed 25 m in front of the goal and a semi-high-speed video camera (EX-F1, Casio, 300 fps), which filmed each kick, was placed 0.5 m to the left of the kicking robot. For this reason, the rotation of the soccer ball launched by the kicking robot was measured using a high-speed camera set up next to the robot. The computed results are only for non-rotating balls with less than one rotation. The location at which the flying ball, launched by the kicking robot, struck the net of the goal was set as the point of impact of the ball. A comparison was made of the points of impact for each soccer ball face.

The launch conditions for the kicking robot were set to an initial velocity of 30 m/s and no rotation (less than one rotation) of the ball. The experiment was repeated several times. To minimise the kicking robot error that occurred due to the kicking sequence, the ball orientation sequence was changed each time. Thus, kicks to faces A, B, and C were measured sequentially, in that order. Moreover, the location where the ball, launched by the kicking robot, struck the net was filmed with a camera installed 25 m in front of the goal, and the ball’s point of impact was measured. For the data analysis, the ball was launched 20 times for each ball orientation, and its point of impact was analysed for each orientation.

### D-PIV tests

**Figure 10:**
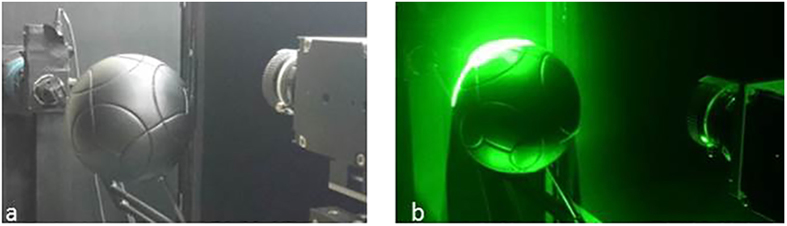
Setup for soccer ball PIV measurement (a) Before run, (b) During run.

The 2D-PIV measurements were carried out on the centreline of the soccer ball. Micro-droplets with diameters of 1 μm were generated by an aerosol generator (PivPart40, PivTec), and were introduced into the flow by a sirocco fan in the wind tunnel. A high-repetition-rate pulsed ND:Yag laser (LDP-100MQG, Lee Laser) was used to illuminate the microdroplet particles. A high-speed camera (Memrecam GX-8, Nac) was used to record tiff images at a sampling frequency of 1000 Hz. The air flow velocity was set to 30 m/s. Figure 10(a) shows an image taken before the run, while Fig. 10(b) shows an image taken during the run.

A time-resolved PIV system can obtain the traveled distances of the particles using double-pulsed laser over a brief period of time. By dividing this traveled distance by the double-pulse time intervals, the velocity vector can be obtained. However, in the PIV measurements, velocity vectors are not obtained for individual particles but for particle groups in the measurement range. In other words, the displacement of the intensity patterns formed by the particle groups in the measurement range is tracked. The similarity of the local intensity patterns between images over two times are obtained through cross-correlation, and the traveled distance is determined from these peak locations. This traveled distance is divided by the time interval to obtain the group velocity.

For the present experiment, a double-pulsed laser was used at 30-μs intervals. During this time, particles moved over approximately 3–5 pixels. The measurement range was taken as 32 pixels × 32 pixels, and measurements were taken over 3 s with a double-pulse frequency of 1 KHz. One pixel is approximately 0.2 mm.

**Figure 11:**
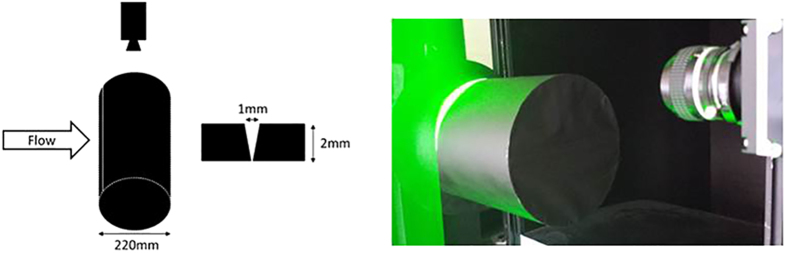
Experimental setup for PIV measurement of cylinder.

In addition, we performed PIV tests using a cylinder to confirm the effect of the grooves between panels (Fig. 11). The width of the groove was set to 1 mm and the depth to 2 mm. The cylinder was rotated between 0° and 150° in 15° increments, and the aerodynamic forces were measured at an air flow velocity (*U*) of 24 m/s to examine how the aerodynamic forces changed as the orientation was adjusted. The forces acting on the cylinder were measured by a sting-type 3-component force detector (LMC-3531-50NS, Nissho Electric Works). The aerodynamic forces measured in this test were converted into a lift coefficient (*Cl*), as given by Eq. (2).

## Additional Information

**How to cite this article**: Hong, S. *et al.* Visualization of air flow around soccer ball using a particle image velocimetry. *Sci. Rep.* **5**, 15108; doi: 10.1038/srep15108 (2015).
